# LncRNA MEG8 sponging miR-181a-5p contributes to M1 macrophage polarization by regulating SHP2 expression in Henoch-Schonlein purpura rats

**DOI:** 10.1080/07853890.2021.1969033

**Published:** 2021-09-03

**Authors:** Mingyu Jiang, Jicheng Dai, Mingying Yin, Chunming Jiang, Mingyong Ren, Lin Tian

**Affiliations:** a Department of Pediatrics, The First Affiliated Hospital of Harbin Medical University, Harbin, P. R. China; b Department of Pediatrics, The Fourth Affiliated Hospital of Harbin Medical University, Harbin, P. R. China; c Department of Pathology, The First Affiliated Hospital of Harbin Medical University, Harbin, P. R. China

**Keywords:** Henoch–Schonlein purpura, maternally expressed gene 8, miR-181a-5p, macrophage polarization, suppressor of SH2 domain-containing tyrosine phosphatase 2

## Abstract

**Background:**

Long noncoding RNAs (LncRNAs) are regulatory molecules that play important roles in various biological and pathological processes. Herein, we aimed to explore whether maternally expressed gene 8 (MEG8) promotes M1 macrophage polarization among Henoch-Schonlein purpura (HSP) rats, and to investigate the underlying mechanism.

**Methods:**

Relative mRNA expression of MEG8, miR-181a-5p and suppressor of SH2 domain-containing tyrosine phosphatase 2 (SHP2) were examined using quantitative reverse transcription polymerase chain reaction. Furthermore, expression of SHP2 and the Janus kinase 2/signal transducer and activator of transcription 3 (JAK2/STAT3) pathway-related proteins was identified using western blot. Luciferase activity assay was conducted to evaluate whether miR-181a-5p could bind to MEG8 or SHP2. The macrophage phenotype was determined using flow cytometry and enzyme-linked immunosorbent assay.

**Results:**

We observed macrophage polarization towards the M2 phenotype in the peripheral blood of HSP rats. Furthermore, MEG8 and SHP2 expression were down-regulated, while miR-181a-5p was up-regulated in monocyte-derived macrophages from the HSP rats compared to the control group. Furthermore, MEG8 functioned as a sponge for miR-181a-5p in order to facilitate SHP2 expression. Moreover, miR-181a-5p mimic and SHP2 knockdown significantly reversed the MEG8 overexpression-mediated suppression of JAK2/STAT3 signalling, and promotion of M1 polarization.

**Conclusions:**

The lncRNA MEG8 sponged miR-181a-5p, which contributes to M1 macrophage polarization by regulating SHP2 expression in HSP rats.

**Key Messages**
- LncRNA MEG8 downregulation and M2 polarization in Henoch Schonlein purpura rats.
- MEG8 upregulation enhances M1 polarization and suppresses JAK2/STAT3 pathway.
- MEG8 sponges miRNA-181a-5p to regulate SHP2 expression.
- MiRNA-181a-5p upregulation reverses lncRNA MEG8-mediated enhancement of M1 polarization and inhibition of JAK2/STAT3 pathway.
- SHP2 downregulation reverses lncRNA MEG8-mediated enhancement of M1 polarization and inhibition of JAK2/STAT3 pathway.

## Introduction

Henoch-Schonlein purpura (HSP) is a rare disorder that involves swollen small blood vessels of the skin, joints, digestive system and kidneys, and to a lesser extent the central nervous system (CNS) and lungs [1]. HSP typically affects children, and may continue into adulthood. The prevalence of HSP peaks in children aged 3–10 years, and it affects 10–20 per 100,000 children every year. HSP tends to increase the risk of long-term renal impairment among adult patients [2]. Although the exact aetiology of HSP is still unknown, the genetic, environmental and immunological factors are likely to be involved [3].

Macrophages are common innate immune cells that have crucial roles in many physiological and pathological processes, including tissue development, inflammatory responses, tissue remodelling, host defense and homeostasis [4]. The activated macrophages can be polarized into the classically activated type 1 (M1) or the alternatively activated type 2 (M2) depending on their differentiation state. M1-like polarized macrophages are usually triggered by granulocyte-macrophage colony stimulating factor, and exhibit high levels of proinflammatory and phagocytic activities. CD64+ and CD80+ are the most suitable biomarkers to characterize these macrophages, due to their ability to regulate acute inflammation. These surface markers are known to secrete proinflammatory cytokines, including IFN-γ, TNF-α, IL-1, -6 and -12 [5]. On the other hand, M2-like polarized macrophages are thought to be involved in fungal, helminthic and parasitic infections, and can be activated by Th2 cells. IL-4, -10, -13, macrophage colony-stimulating factor (M-CSF) and a combination of these factors can enhance the polarization of macrophages towards M2 phenotype. CD206+ and CD209+ are the best surface markers for the characterization of M2-like macrophages [6].

Micro RNA (miRNA) is short noncoding RNA that comprises approximately 22 nt, which plays essential roles in regulating inflammatory and immune responses. By binding to specific sites within the 3′-untranslated region (UTR) of target mRNA, miRNAs are able to negatively modulate gene expression via translational repression or mRNA degradation. It has been reported that miRNAs can regulate the development and function of macrophages [7]. Long noncoding RNA (lncRNA) is a type of nonprotein-coding transcript with lengths exceeding 200 nt, and is associated with various physiological and pathological processes. Overall, lncRNAs mediate transcriptional and post-transcriptional regulation, which is a crucial underlying mechanism of epigenetic programming [8]. A large number of studies have suggested that lncRNAs are involved in the activation and polarization of macrophages [9]. In addition, lncRNA MEG8 can act as a regulator in atherosclerotic lesions, renal impairment, cancer occurrence and epithelial-mesenchymal transition [10]. However, its role in HSP remains largely unclarified.

Through the use of bioinformatic tools (starBase and TargetScan), we identified that lncRNA MEG8 harboured putative binding sites of miRNA-181a-5p, and SH2 domain-containing tyrosine phosphatase 2 (SHP2) could be the putative target of miRNA-181a-5p. Previous research has shown that SHP2 mediates the polarization of M1 and M2 macrophages by negatively regulating Janus kinase 2/signal transducer and activator of transcription 3 (JAK2/STAT3) pathway [11]. Thus, we speculate that lncRNA MEG8 may sponge miRNA-181a-5p to regulate SHP2 expression and inhibit JAK2/STAT3 pathway, thus promoting the polarization of M1 macrophages in HSP.

## Materials and methods

### Ethical approval

The animal experimentation was approved by the Institutional Animal Care and Use Committee of Harbin Medical University, and was performed in compliance with the guidelines for care and use of laboratory animals.

### Animal models

Wistar rats (10–12 weeks old, weighing 250–300 g) were selected for the study. The rats were supplied by the Animal Research Centre of Harbin Medical University (China). To establish an HSP model, the animals were intraperitoneally sensitized with 1 mL of normal saline containing 10 mg alum (Sigma-Aldrich, USA) and 1 mg ovalbumin (Sigma-Aldrich, USA) once a week for two weeks. Then, 15 mg/kg of ovalbumin in normal saline was given via the external jugular, and 1 mL of 0.3% ovalbumin in normal saline was intradermally injected at five different sites. An equivalent amount of normal saline was given to the control rats [12]. After anaesthesia with 40 mg/kg pentobarbital sodium (Sigma-Aldrich, USA), blood specimens were withdrawn from the rats for subsequent analysis. Finally, by adhering to the 3 R criteria in animal experiments: "reduce, replace, and optimize", all rats were given an intraperitoneal injection of 150 mg/kg pentobarbital sodium for euthanasia.

### Preparation of rat monocyte-derived macrophages (RMDMs)

RMDMs were isolated and cultured according to previously reported methods [13]. First, the isolation of peripheral blood mononuclear cells (PBMCs) was performed by Ficoll-Paque density gradient centrifugation (Solarbio Life Sciences, China). Then, anti-CD14 magnetic beads (Miltenyi Biotec, USA) were used to purify monocytes from the isolated PBMCs as per the manufacturer’s protocol. After culturing in RPMI 1640 (Sigma-Aldrich, USA) supplemented with 10% FBS (Sigma-Aldrich, USA) for 7 days, macrophages differentiated from monocytes the purified monocytes in the presence of 50 ng/mL M-CSF (Sigma-Aldrich, USA) for 7 days. Half-medium changes were performed every 3 days, unless otherwise specified.

### Flow cytometric analysis and ELISA detection

The surface markers of RMDMs were quantified by flow cytometry as described previously [14]. First, the RMDMs were stained with anti-CD206-MR5D3 (Novus Biologicals, USA) and anti-CD80-PE (Thermo Fisher Scientific, USA). After fixing, the RMDMs were subjected to flow cytometric analysis (Accuri C6; Becton Dickinson, USA). FlowJo software (Tree Star Inc., USA) was used for data analysis. The levels of TGF-β, IFN-γ, IL-4 and −12 in RMDMs from HSP and normal rats were determined using the corresponding ELISA kits (R&D Systems, USA) as per the manufacturer’s protocols.

### Real-time quantitative polymerase chain reaction (RT-qPCR)

RNA extraction was performed using the RNeasy Plus Mini Kit (Invitrogen, USA) as per the manufacturer’s protocol. Next, the PrimeScript RT Master Mix kit (TaKaRa Biotechnology, Japan) was used for cDNA synthesis. The RT-qPCR assay was prepared with 20 µL reaction mixture consisting of cDNA (1 µL), 2 × All-in-one qPCR Mix (10 µL; TaKaRa Biotechnology, Japan), 2 mmol/L forward and reverse primers (1 µL each) and of nuclease-free water (6 µL). The RT-qPCR cycles were as follows: initial denaturation at 95 °C for 10 min, followed by 40 cycles 95 °C for 10 s, 60 °C for 20 s, and 72 °C for 15 s. GAPDH (TaKaRa Biotechnology, Japan) was used as an internal control. The expression levels of miRNA, lncRNA and mRNA were quantified through the 2^−ΔΔCt^ method [15]. The expression levels of miRNA-181a-5p and lncRNA MEG8 were normalized to 18S rRNA, while the mRNA expression of SHP2 was normalized to GAPDH. The primer sequences are shown in Table 1.

**Table 1.** Primers used in the study.

Genes	Primer sequences	Annealing temperature (°C)	PCR product (bp)
GAPDH	sense: 5′-TCGCCAGCCGAGCCACAT-3′	60	149
	anti-sense: 5′-GGAACATGTAAACCATGTAGTTG-3′		
MEG8	sense: 5′-CATCTAGACCCGTAACGCCC-3′	60	135
	anti-sense: 5′-CATTCCTCGGGTGTGGAGAC-3′		
miR-181a-5p	sense: 5′-CGGGCAACATTCAACGCTGT-3′	60	102
	anti-sense: 5′-GTGCAGGGTCCGAGGTATTC-3′		
SHP2	sense: 5′-CCGCAGATTCAGGGATTACT-3′	60	92
	anti-sense: 5′-CTTGGAAACGGACCAGTTCT-3′		
18S	sense: 5′-AGGGTTCGATTCCGGAGAGG-3′	60	110
	anti-sense: 5′-CAACTTTAATATACGCTATTGG-3′		
iNOS	sense: 5′-GCCAGAGAGCCAGGAGCA-3′	60	135
	anti-sense: 5′-ACACAGATAAACTTGGTCTTCAGGTATG-3′		
IL-1β	sense: 5′-CGAACTTCATTCAACAATGA-3′	60	109
	anti-sense: 5′-GTTTAGCCTAAGAGGTATAG-3′		
TNF-α	sense: 5′-ATACAGATATAGGGATTACA-3′	60	98
	anti-sense: 5′-CTTGGATTCGGACCAGTTAG-3′		
CXCL10	sense: 5′-AGATATAGGGATTACAGGATT-3′	60	91
	anti-sense: 5′-ACCAGTTAATTCGGATCTGAC-3′		
CCR7	sense: 5′-CCGCAGATTACTGATTCAGG-3′	60	101
	anti-sense: 5′-CTTGGCCAGTTCTAAACGGA-3′		
IL-10	sense: 5′-TTCAGGCAGAGAGCCTTACT-3′	60	112
	anti-sense: 5′-AGTTCTTGGAACTACGGACC-3′		
CCL1	sense: 5′-AAATCTGCAAACGAGAATGC-3′	60	118
	anti-sense: 5′-CACAGGATGTTCCCCAGATT-3′		
CCL2	sense: 5′-AGAATTCCTTAGAGATTCTT-3′	60	127
	anti-sense: 5′-CAACGATACTATTACTATACGA-3′		
CCL17	sense: 5′-GCAGCATAGCAGATGTGAA-3′	60	129
	anti-sense: 5′-TGAACGCTCCAGGATTTA-3′		
Arg-1	sense: 5′-CGTCAAGTGCCAGCCCTCA-3′	60	133
	anti-sense: 5′-TACGCCTTCCCGTCTCCT-3′		

GAPDH: glyceraldehyde 3-phosphate dehydrogenase; MEG8: maternally expressed gene 8; SHP2: the Src homology phosphotyrosylphosphatase 2; iNOS: inducible NOS; IL-1β: interleukin-1β; TNF-α: tumour necrosis factor-α; CXCL10: CXCchemokineligand-10; CCR7: C–C motif chemokine receptor 7; IL-10: interleukin-10; CCL: C–C motif chemokine ligand; Arg-1: arginase 1.

### Detection of macrophage phagocytosis

The identified M1 and M2 macrophages in each group were seeded on a 6-well plate at a density of 2 × 10^5^ per well. Each group had two double holes. Upon reaching 80% confluence, the cells were digested and incubated with 200 μL of 10% FBS medium and 5 μL of red zymosan for 2 h. After centrifugation, the supernatant was discarded, and the suspended cells were rinsed three times with 500 μL phagocytosis assay buffer prior to flow cytometric analysis.

### Immunoblotting

The protein expression in RMDMs was identified by immunoblotting. Briefly, the cells were lysed with RIPA lysis buffer (Beyotime Biotechnology, China). The protein content was detected using the BCA assay kit (Beyotime Biotechnology, China). Subsequently, equivalent amounts of total protein were separated through SDS-PAGE (Beyotime Biotechnology, China), and transferred onto PDVF membranes (Beyotime Biotechnology, China). The membranes were blocked with 5% skimmed milk, and then added with primary antibodies. After incubation at 4 °C overnight, the corresponding horseradish peroxidase-conjugated secondary antibodies were added. The protein bands were visualized with ECL detection reagent (Seven Seas Biotechnology, China). Data analysis was conducted by Gel-Pro-Analyzer (Media Cybernetics, USA).

### Plasmid construction and cell transfection

To obtain a pcDNA3.1-MEG8 vector, lncRNA MEG8 sequences were synthesized and subcloned into the expression vector pcDNA3.1 (Invitrogen, USA). An empty pcDNA3.1 vector was employed as the control group. Cell transfection was conducted with Lipofectamine 2000 (Invitrogen, USA) as per manufacturer’s protocol. To knockdown SHP2 and lncRNA MEG8, the cells were transfected with SHP2 small interfering RNA (si-SHP2), si-MEG8 and scramble controls (GenePharma, China) using the Lipofectamine RNAiMAX Reagent (Invitrogen, USA). Meanwhile, hsa-miRNA-181a-5p mimic, hsa-miRNA-181a-5p inhibitor, mimic negative control (NC) and NC inhibitor (GenePharma, USA) were transfected with Lipofectamine 2000 (Invitrogen, USA) as per the manufacturer’s protocol. After 48 h of transfection, the knockdown or overexpression efficiency was verified by RT-qPCR assay [16].

### Luciferase reporter assay

PCR amplification was conducted on the fragments of SHP2 3′-UTR and lncRNA MEG8 containing WT or MUT miRNA-181a-5p. These fragments were then inserted into the luciferase reporter vector pMIR-REPORT (Ambion, USA), which were named as SHP2-WT, SHP2-MUT, lncRNA MEG8-WT and lncRNA MEG8-MUT. Subsequently, the cells were co-transfected with 200 ng luciferase reporter construct, 25 ng pRL-TK plasmid, and 20 µM miRNA-181a-5p mimic or mimic NC using the Lipofectamine 2000. After 24 h of transfection, the luciferase activity of each sample was examined using the luciferase reporter assay system (Promega, USA).

### Statistical analyses

All *in vitro* assays were repeated three times. SPSS v20.0 software was used to perform all statistical tests. The difference between the two variables was compared using the unpaired Student’s t-test. One-way ANOVA was employed to analysis the differences among multiple groups. All data were expressed as mean ± standard deviation (SD). Level of significance was set as *p*<.05.

## Results

### LncRNA MEG8 downregulation and M2 polarization in HSP rats

**Figure 1.**
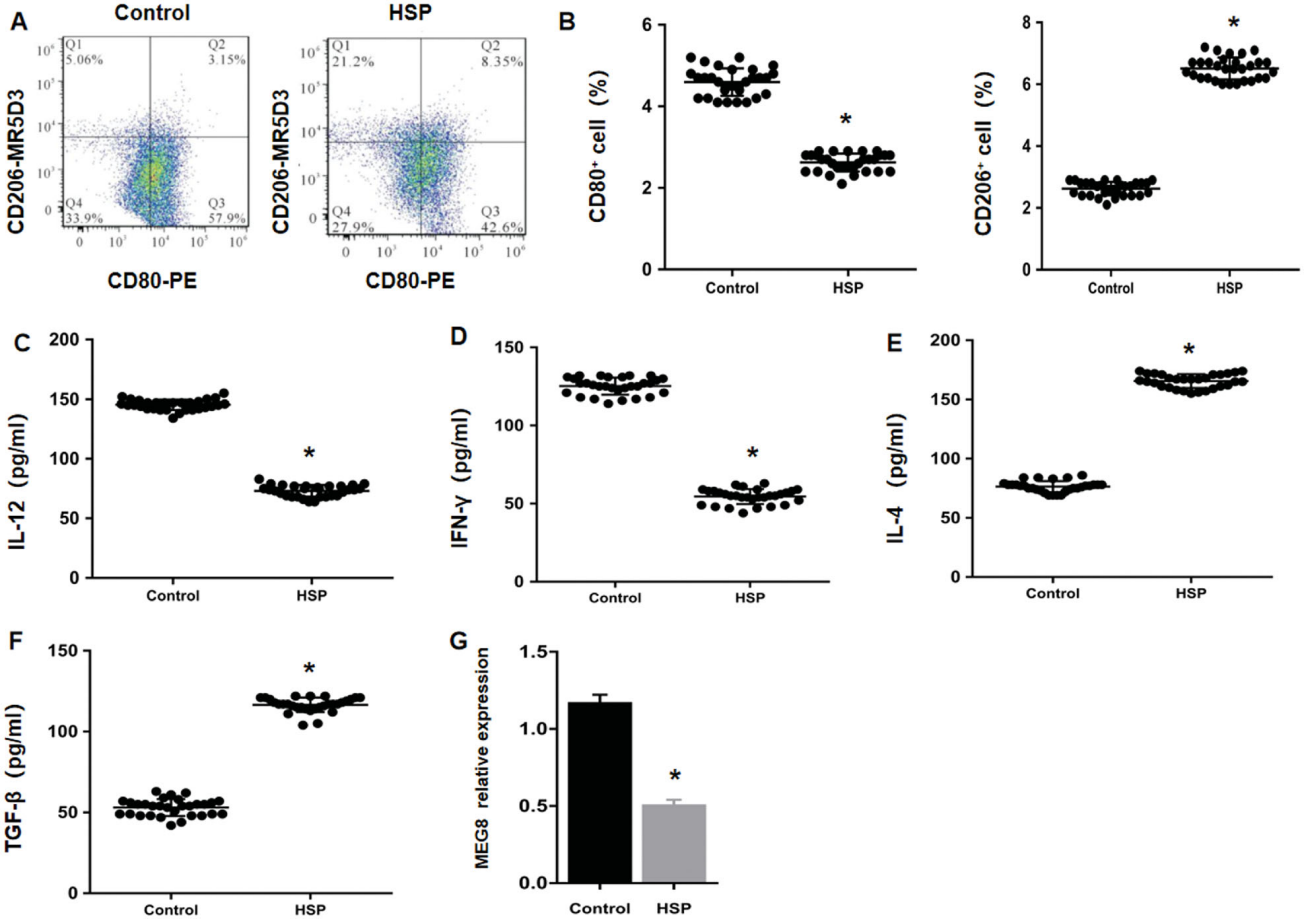
Low MEG8 expression and M2 polarisation in Henoch Schonlein purpura. (A) Representative scatter plots demonstrate peripheral blood levels of CD80 and CD206, evaluated using flow cytometry. (B) The histogram shows statistical analysis for the proportion of CD80+ and CD206+ macrophages. Concentrations of IL-12 (C), IFN-γ (D), IL-4 (E), and TGF-β (F) using ELISA, and relative MEG8 expression examined using qRT-PCR (G) in rat monocyte-derived macrophages from Henoch Schonlein purpura (*n* = 25) and control subjects (*n* = 23). *p<.05 compared to the control group. MEG8: maternally expressed gene 8; IFN-γ: Interferon γ; TGF-β: transforming growth factor β; ELISA: enzyme-linked immunosorbent assay; qRT-PCR: quantitative reverse transcription polymerase chain reaction.

Flow cytometric analysis revealed that the percentages of the M1 (CD80^+^) and M2 (CD206^+^) macrophage markers were remarkably decreased and increased, respectively, in HSP rats compared with normal rats (Figure 1(A,B)). Moreover, the results of enzyme-linked immunosorbent assay (ELISA) showed that the levels of the M1 (IFN-γ and IL-12) and M2 (TGF-β and IL-4) macrophage cytokines were markedly decreased and increased, respectively, in the RMDMs of HSP group compared with the control group (Figure 1(C–F)). These findings indicate that the macrophages HSP rats are polarized from M1 to M2 phenotypes. RT-qPCR data indicated that the expression of lncRNA MEG8 was much lower in HSP rats than in normal rats (Figure 1(G)).

**Figure 2.**
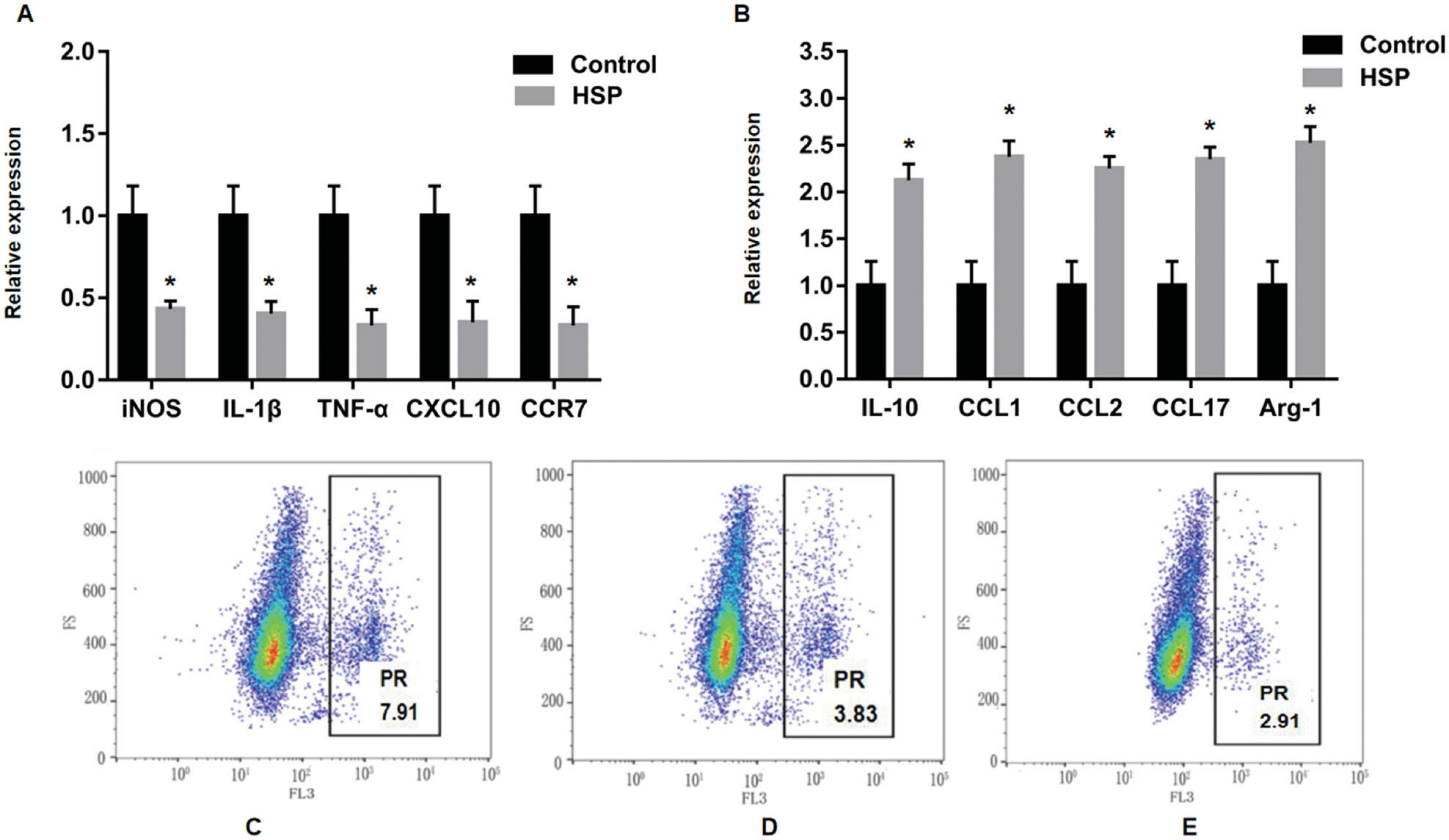
(A) Functional markers of M1 macrophages (iNOS, IL-1β, TNF-α, CXCL10, and CCR7) and (B) M2 macrophages (IL-10, CCL1, CCL2, CCL17, and Arg-1) using quantitative reverse transcription polymerase chain reaction. In addition, the PR (phagocytic rate) of M1 macrophage (C), M2 macrophages (D) and the control group (E) was determined using flow cytometry. *p<.05 compared to the control group. iNOS: inducible NOS; IL-1β: interleukin-1β; TNF-α: tumour necrosis factor-α; CXCL10: CXCchemokineligand-10; CCR7: C-C Motif Chemokine Receptor 7; IL-10: interleukin-10; CCL: C-C Motif Chemokine Ligand; Arg-1: Arginase 1.

Furthermore, the expression levels of other M1 macrophage-related cytokines (iNOS, IL-1β, TNF-α, CXCL10, and CCR7) were remarkably lower, while those of other M2 macrophage-related cytokines (IL-10, CCL1, CCL2, CCL17, and Arg-1) were significantly higher in the RMDMs of HSP rats compared to normal rats (Figure 2(A,B)), which were consistent the above findings. The phagocytosis of M1 and M2 macrophages was assessed by flow cytometry. The phagocytic rate of M1 macrophages was relatively higher than that of M2 macrophages in HSP rats, and these values were significantly increased compared with those in normal rats (Figure 2(C–E)). Our results demonstrated that M1 macrophages were activated, phagocytosis was enhanced, and the surrounding tissues were injured, thus aggravating the process of Henoch-Schonlein purpura.

### MEG8 upregulation enhances M1 polarization and suppresses JAK2/STAT3 pathway

**Figure 3.**
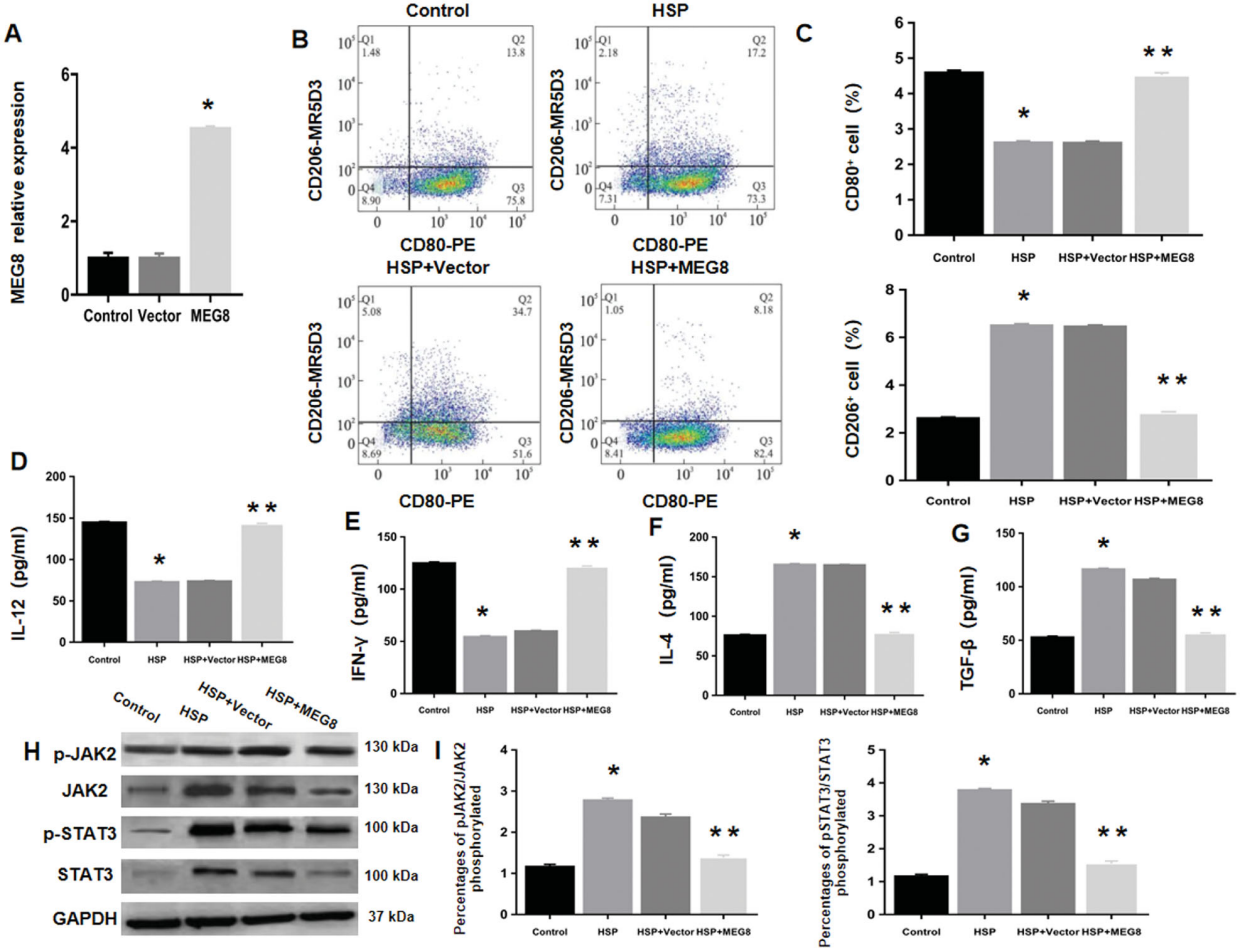
MEG8 overexpression promoted M1 polarization and suppressed JAK2/STAT3 signalling. (A) Overexpression efficiency of MEG8 in monocyte-derived macrophages from Henoch Schonlein purpura rats was validated using qRT-PCR. *p<.05 comparted to the vector group. Representative scatter plots showing levels of CD80 and CD206 evaluated using flow cytometry. (B) Statistical analysis for the proportions of CD80+ and CD206+ macrophages. (C) Concentrations of IL-12 (D), IFN-γ (E), IL-4 (F), and TGF-β (G) using ELISA, and expression of JAK2/STAT3 pathway-related proteins using western blot analysis (H–I), in control rats-derived macrophages (Control), Henoch Schonlein purpura rats-derived macrophages (HSP), empty vector-transfected Henoch Schonlein purpura macrophages (HSP + Vector), and MEG8-overexpressing Henoch Schonlein purpura macrophages (HSP + MEG8). *p<.05 compared to the control group; **p<.05 compared to the HSP + vector group. GAPDH: glyceraldehyde 3-phosphate dehydrogenase; JAK2: Janus kinase 2; STAT3: signal transducer and activator of transcription 3; ELISA: enzyme-linked immunosorbent assay; qRT-PCR: quantitative reverse transcription polymerase chain reaction.

Next, we explored the roles of lncRNA MEG8 in macrophage polarizations. lncRNA MEG8 was overexpressed in the RMDMs of HSP rats by transfecting with pcDNA3.1-MEG8 (Figure 3(A)). Flow cytometric analyses demonstrated that lncRNA MEG8 upregulation markedly increased and decreased the percentages of CD80+ and CD206+ macrophages, respectively, in HSP rats compared to the vector control group (Figure 3(B,C)). In addition, the ELISA results showed that lncRNA MEG8 upregulation markedly increased the levels of IFN-γ and IL-12, while decreased those of TGF-β and IL-4 in HSP macrophages compared to the vector control group (Figure 3(D–G)). These findings suggest that lncRNA MEG8 upregulation reverses the polarization of RMDMs from M2 to M1 phenotypes [17].

In addition, we examined the role of JAK2/STAT3 signal transduction in regulating macrophage polarization. The results demonstrated that the phosphorylation levels of JAK2 and STAT3 were remarkably higher in the RMDMs of HSP rats compared with normal rats (Figure 3(H,I)). This indicates JAK2/STAT3 pathway is associated with macrophage polarization in HSP rats. Furthermore, lncRNA MEG8 upregulation reduced the high phosphorylation levels of JAK2 and STAT3 in HSP rats (Figure 3(H,I)), indicating that JAK2/STAT3 pathway was suppressed by lncRNA MEG8 upregulation.

### MEG8 sponges miRNA-181a-5p to regulate SHP2 expression

**Figure 4.**
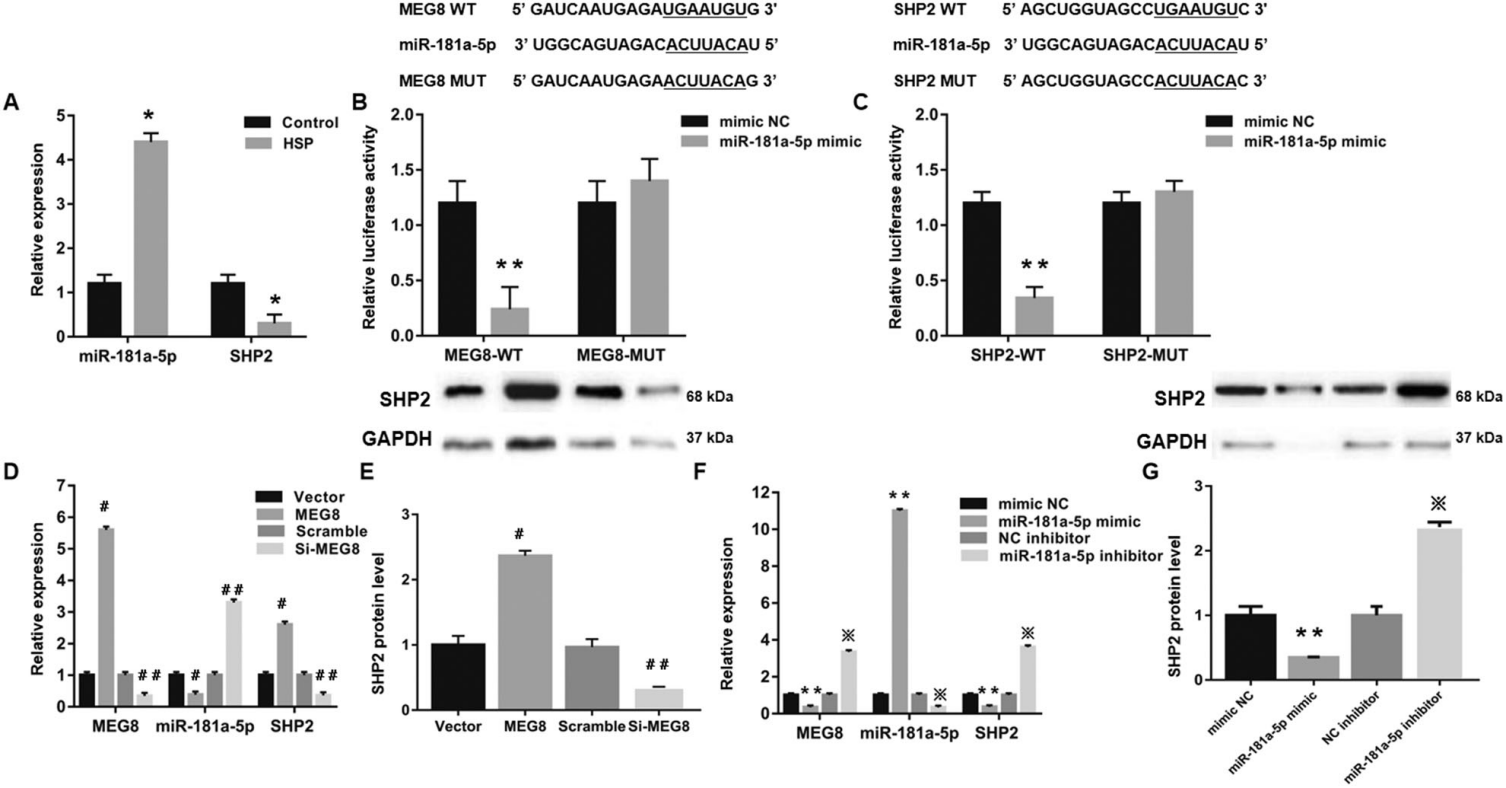
MEG8 acts as a competitive endogenous RNA by sponging miR-181a-5p to facilitate SHP2. (A) Expression of MEG8 and SHP2 in control and Henoch Schonlein purpura macrophages was examined using qRT-PCR. *p<.05 compared to the control group. (B) Luciferase reporter assay validated the direct binding between MEG8 and miR-181a-5p. **p<.05 compared to the MEG8 WT + mimic NC group. (C) Luciferase reporter assay suggests that 3′-UTR of SHP2 is directly targeted by miR-181a-5p. **p<.05 compared to the SHP2 WT + mimic NC group. Relative expression of MEG8 and miR-181a-5p, SHP2 mRNA expression using qRT-PCR (D), and SHP2 protein expression using western blot analysis (E) in Henoch Schonlein purpura macrophages transfected with pcDNA3.1-MEG8 vector, control empty vector, si-MEG8, or scramble siRNA. ＃*p*<.05 compared to the vector group and ＃＃*p*<.05 compared to the scramble group. Relative expression of MEG8 and miR-181a-5p, SHP2 mRNA expression using qRT-PCR (F) and SHP2 protein expression using western blot analysis (G) in Henoch-Schonlein purpura macrophages transfected with miR-181a-5p mimic, mimic NC, miR-181a-5p inhibitor, or NC inhibitor. **p<.05 compared to the mimic NC group; ※*p*<.05 compared to the inhibitor NC group. mRNA: messenger RNA; NC: negative control; qRT-PCR: quantitative reverse transcription polymerase chain reaction; siRNA: small interfering RNA; SHP2: the Src homology phosphotyrosylphosphatase 2; UTR: untranslated region; WT: wild type.

Next, we elucidated the mechanisms by which lncRNA MEG8 enhances M1 polarization and suppresses JAK2/STAT3 pathway. Notably, the mRNA expression of SHP2 was remarkably decreased in HSP macrophages compared with control macrophages, which was consistent with the expression pattern of lncRNA MEG8. On the contrary, the expression of miRNA-181a-5p was much higher in HSP macrophages than in control macrophages (Figure 4(A)).

Then, we evaluated the relationships among SHP2, miRNA-181a-5p and lncRNA MEG8. Results from the luciferase reporter assay demonstrated that the luciferase activity of lncRNA MEG8-WT reporter was dramatically lower in miRNA-181a-5p mimic group than in the mimic NC group. On the other hand, there was no obvious difference in the luciferase activity of lncRNA MEG8-MUT reporter between the two groups. These data verify a direct interaction between lncRNA MEG8 and miRNA-181a-5p (Figure 4(B)). Moreover, it was found that the luciferase activity of cells co-transfected with miRNA-181a-5p mimic and WT SHP2 3′-UTR luciferase reporter plasmids (Figure 4(C)). This suggests that miRNA-181a-5p directly targets the 3′-UTR of SHP2. Additionally, lncRNA MEG8 upregulation markedly suppressed the expression of miRNA-181a-5p, while elevated the expression of SHP2 at the mRNA and protein levels (Figure 4(D,E)). However, lncRNA MEG8 downregulation showed an opposite effect. Furthermore, miRNA-181a-5p upregulation significantly decreased the expression levels of lncRNA MEG8 and SHP2 in miRNA-181a-5p mimic group, while miRNA-181a-5p knockdown demonstrated the opposite results (Figure 4(F,G)). Altogether, these findings suggest that lncRNA MEG8 sponges miRNA-181a-5p to regulate SHP2 expression.

### MiRNA-181a-5p upregulation reverses lncRNA MEG8-mediated enhancement of M1 polarization and inhibition of JAK2/STAT3 pathway

**Figure 5.**
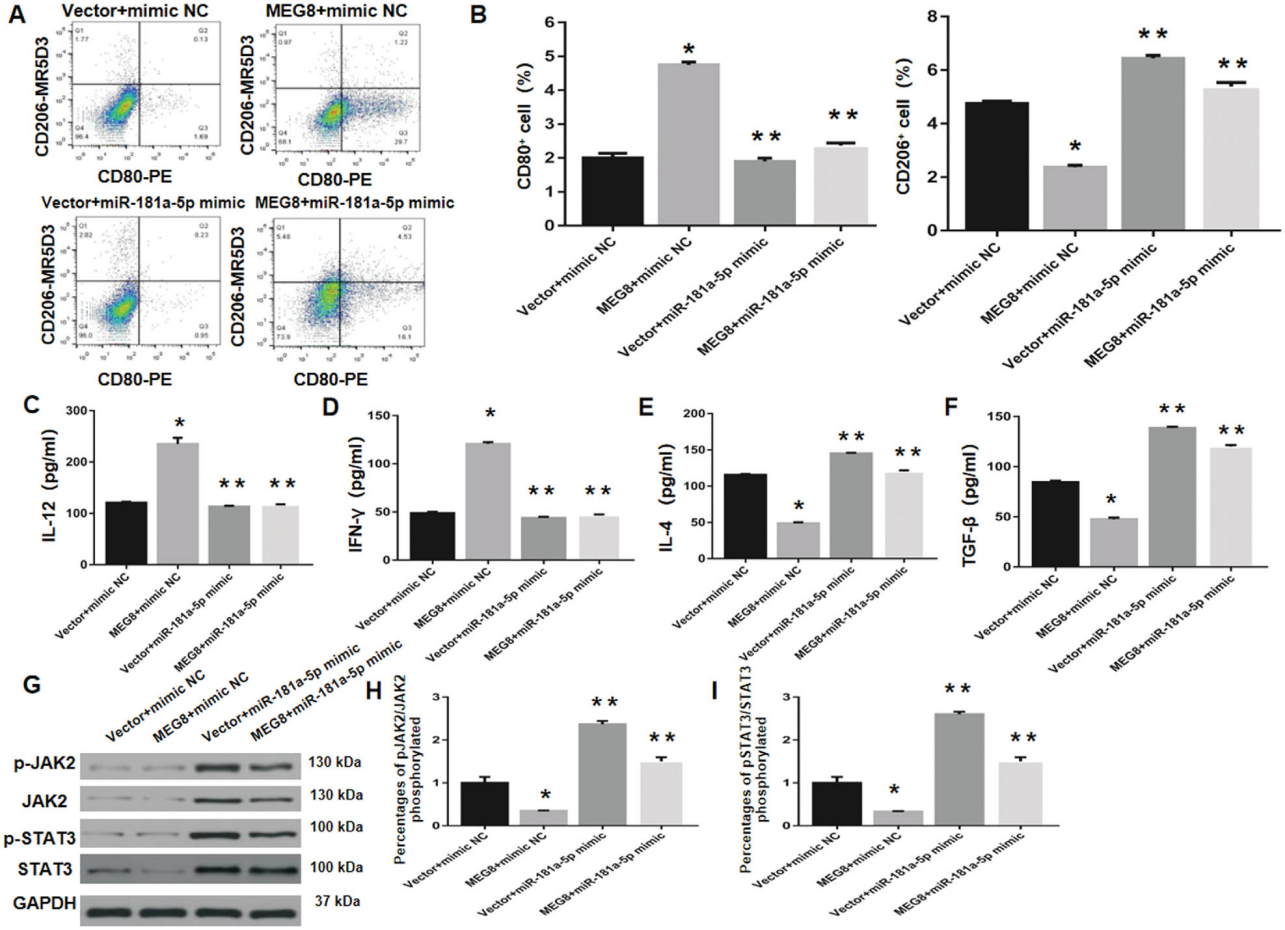
miR-181a-5p overexpression reverses MEG8-mediated promotion of M1 polarization and activation of JAK2/STAT3 signalling. (A) Representative scatter plots demonstrating levels of CD80 and CD206, evaluated using flow cytometry. (B) Statistical analysis for the percentages of CD80+ and CD206+ macrophages. Concentrations of IL-12 (C), IFN-γ (D), IL-4 (E), and TGF-β (F) using ELISA, and expression of JAK2/STAT3 pathway-related proteins using western blot analysis (G, H, I), in Henoch Schonlein purpura macrophages that were transfected with specific plasmids, including pcDNA3.1-MEG8 vector, control empty vector, miR-181a-5p mimic, or mimic NC. *p<.05 compared to the vector + mimic NC group and **p<.05 compared to the MEG8 +mimic NC group. MEG8: maternally expressed gene 8; IFN-γ: Interferon γ; TGF-β: transforming growth factor β; ELISA: enzyme-linked immunosorbent assay.

Flow cytometric analysis revealed that the percentages of CD80+ and CD206+ macrophages were obviously decreased and increased, respectively, in lncRNA MEG8 + miRNA-181a-5p mimic group compared to lncRNA MEG8 + mimic NC group (Figure 5(A,B)). Moreover, the ELISA results indicated that the levels of IFN-γ and IL-12 were remarkably decreased, while those of TGF-β and IL-4 were increased, in lncRNA MEG8 + miRNA-181a-5p mimic group compared to lncRNA MEG8 + mimic NC group (Figure 5(C–F)). These data indicate that miRNA-181a-5p upregulation can reverse lncRNA MEG8-mediated enhancement of M1 polarization. In addition, the phosphorylation levels of JAK2 and STAT3 were markedly higher in lncRNA MEG8 + miRNA-181a-5p mimic group than in lncRNA MEG8 + mimic NC group (Figure 5(G–I)). This suggests that miRNA-181a-5p upregulation can reverse lncRNA MEG8-mediated inhibition of the JAK2/STAT3 pathway.

### SHP2 downregulation reverses lncRNA MEG8-mediated enhancement of M1 polarization and inhibition of JAK2/STAT3 pathway

Previous research has shown that SHP2 can regulate the polarization of M1 to M2 phenotypes and inhibit JAK2/STAT3 pathway, thus contributing to the aberrant remodelling of fibrotic tissues [18]. Thus, we speculate that SHP2 is involved in lncRNA MEG8-mediated enhancement of M1 polarization and inhibition of the JAK2/STAT3 pathway.

**Figure 6.**
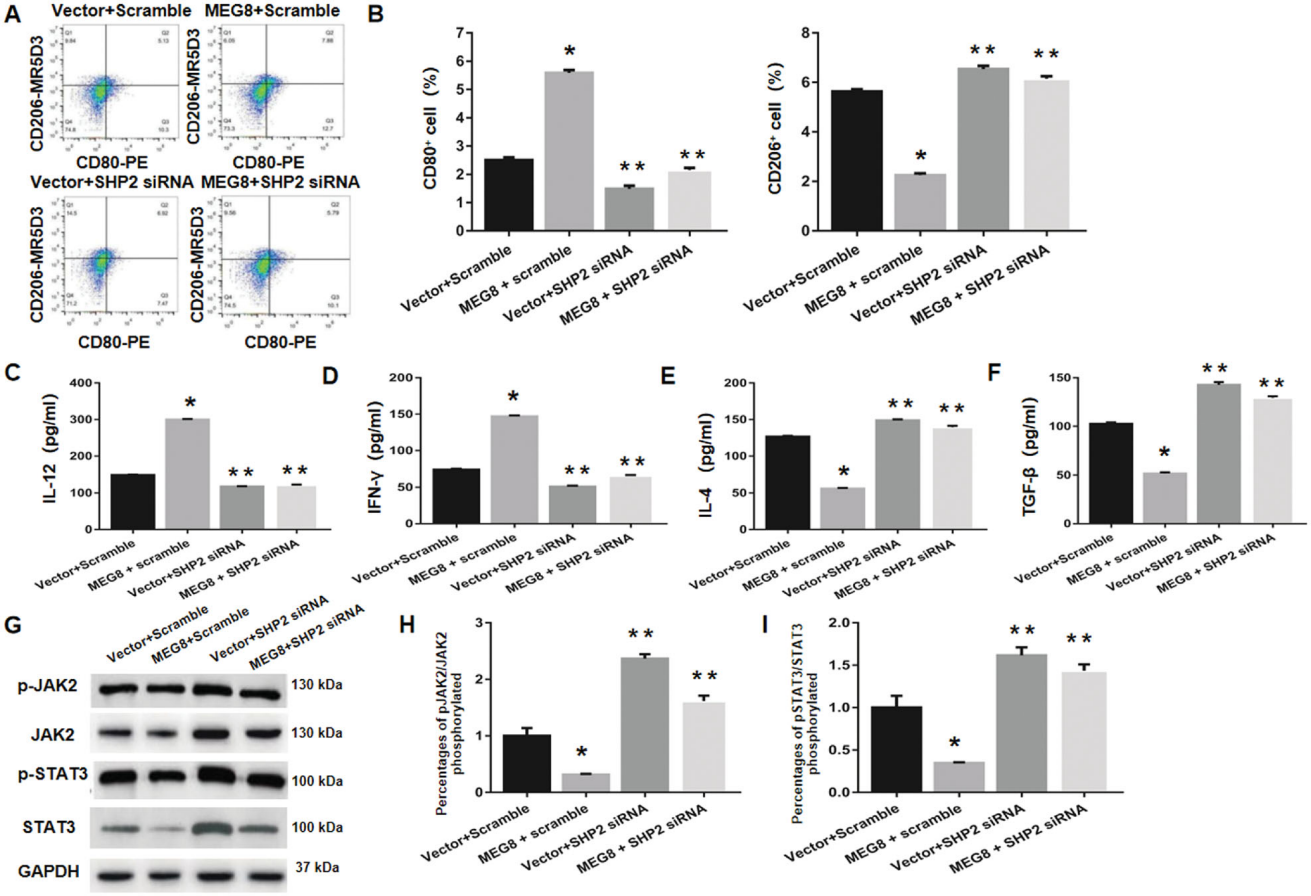
SHP2 knockdown reverses MEG8-mediated promotion of M1 polarization and suppression of JAK2/STAT3 signalling. (A) Representative scatter plots showing levels of CD80 and CD206, evaluated using flow cytometry. (B) Statistical analysis for percentages of CD80+ and CD206+ macrophages. Concentrations of IL-12 (C), IFN-γ (D), IL-4 (E), and TGF-β (F) using ELISA, and expression of JAK2/STAT3 pathway-related proteins using western blot analysis (G, H and I), in Henoch Schonlein purpura macrophages that were transfected with the designated specific plasmids, including pcDNA3.1-MEG8 vector, control empty vector, SHP2 siRNA, or scramble siRNA. *p<.05 compared to the vector + scramble group and **p<.05 compared to the MEG8 + scramble group. MEG8: maternally expressed gene 8; IFN-γ: Interferon γ; TGF-β: transforming growth factor β; ELISA: enzyme-linked immunosorbent assay.

Flow cytometric analysis demonstrated that the percentages of CD80+ and CD206+ macrophages were remarkably decreased and increased, respectively, in lncRNA MEG8 + SHP2 siRNA group compared to lncRNA MEG8 + scramble group (Figure 6(A,B)). Moreover, the ELISA results demonstrated that the levels of IFN-γ and IL-12 were dramatically decreased, while those of TGF-β and IL-4 were increased, in lncRNA MEG8 + SHP2 siRNA group compared to lncRNA MEG8 + scramble group (Figure 6(C–F)). These data suggest that SHP2 downregulation reverses lncRNA MEG8-mediated enhancement of M1 polarization. Furthermore, the phosphorylation levels of JAK2 and STAT3 were markedly higher in lncRNA MEG8 + SHP2 siRNA group than in lncRNA MEG8 + scramble group (Figure 6(G–I)). These results demonstrate that SHP2 downregulation reverses lncRNA MEG8-mediated inhibition of the JAK2/STAT3 pathway.

## Discussion

HSP is characterized by the accumulation of IgA-containing immune complexes at the walls of small blood vessels such as arteriole, capillary and venule, which belongs to a group of nongranulomatous [19]. Inflammation can be classified into different stages, including the initiation, evolution, and resolution of the inflammatory phase. Macrophages play key roles in the initiation and resolution phases of inflammation. These immune cells have been classically described to exist in two states (anti-inflammatory and pro-inflammatory), which are characterized by diametrically opposite phenotypes [20]. Therefore, it is speculated that macrophage differentiation may contribute to the maintenance of tissue homeostasis by altering gene expression and switching cell phenotypes [21].

At present, a growing body of evidence indicates that the functional polarization of macrophages into either M1 or M2 phenotype is a multifactorial process involving a large number of factors, which produces different activation scenarios [22]. Once macrophages adopt a specific phenotype, it will keep changing in order to adapt the new environment [23]. The reversible polarization exhibits certain therapeutic potential, particularly in diseases caused by the imbalance of M1/M2 macrophages [24]. Herein, we found that the macrophages of HSP rats could be polarized from M1 to M2 phenotypes, thereby exhibiting an M2-polarised subtype. Interestingly, lncRNA MEG8 upregulation promotes the polarization of RMDMs from M2 to M1 phenotypes. This indicates that lncRNA MEG8 may exert a potential therapeutic effect on HSP rats.

LncRNAs play a crucial role in modulating gene expression in cells and tissues. Aberrant expression of lncRNAs is associated with the development of many diseases [25]. Several studies have reported that lncRNAs may affect the expression of the target gene via miRNA sponging [26]. It has been reported that lncRNA MEG8 contributes to the epigenetic progression of epithelial-mesenchymal in both pancreatic and lung cancer cells, and suppresses the growth and migration of trophoblast cells and vascular smooth muscle cells [27,28]. Moreover, lncRNA MEG8 downregulates the expression of Smad2, Smad3, Colla1 and α-SMA, and leads to the formation of myofibroblasts, which ultimately alleviates the progression of renal fibrosis [29]. However, the molecular mechanism underlying the therapeutic effect of lncRNA MEG8 on HSP remains largely unclear. In this study, the mRNA expression of SHP2 was found to be decreased in HSP macrophages compared with control macrophages, which was consistent with the downregulated expression of lncRNA MEG8 in HSP rats. On the contrary, the expression of miRNA-181a-5p in HSP macrophages was increased compared to control macrophages. Furthermore, miRNA-181a-5p inhibition led to the upregulated expression of SHP2 and lncRNA MEG8. Collectively, our data indicate that lncRNA MEG8 sponges miRNA-181a-5p to regulate SHP2 expression in HSP rats, which represents a novel lncRNA-miRNA-mRNA regulatory network for this disease.

The present study also discovered that miRNA-181a-5p upregulation and SHP2 downregulation markedly reversed lncRNA MEG8-mediated enhancement of M1 polarization and inhibition of JAK2/STAT3 pathway. The activation of JAK2/STAT3 pathway in HSP macrophages was indicated by the increased phosphorylation levels of JAK2 and STAT3 in HSP group compared to the control group. It has been reported that miRNA-181a-5p can mediate inflammation in dendritic cells and macrophages by targeting c-Fos and IL-β. Moreover, the expression of miRNA-181a-5p is downregulated in the monocytes of obese subjects and cardiovascular disease patients [30]. SHP2 plays a vital role in regulating macrophage proliferation via a direct activation of the MAPK pathway [31]. A previous study has shown that the inactivation of SHP2 can augment IL-4-mediated enhancement of M2 polarization *in vitro* [32]. Another study reveals that SHP2 knockdown in macrophages promotes the association between JAK1 and IL-4Rα and enhances IL-4-mediated activation of the JAK1-STAT6 signalling pathway, which in turn leads to M2 macrophage skewing [33]. Zhao et al. [34] showed that selective inhibition of SHP2 could attenuate inflammation in mice by skewing macrophage polarization towards the M2 phenotype. Quintana and co-workers [35] demonstrated that the inhibition of SHP2 could promote anti-tumour immune responses by directly and selectively depleting M2 macrophages. Zehender et al. [36] reported that SHP2 could regulate fibroblast proliferation and fibrosis by targeting the activation of the TGFβ-induced JAK2/STAT3 signalling pathway. Thus, SHP2-overexpressing fibroblasts are more susceptible to the profibrotic effects of TGFβ, while the stimulatory effects of TGFβ on collagen production and myofibroblast differentiation are decreased in SHP2-inactivated fibroblasts. The pro-fibrotic effect of the TGFβ pathway was also attenuated in the mouse fibroblasts with SHP2 inactivation. A recent study indicated that SHP2 was associated with the polarization of M1 macrophages during Haemophilus influenza infection and the SHP2 inhibition could enhance the polarization of M2 macrophages [37]. Altogether, these findings imply that SHP2 downregulation is an emerging therapeutic strategy for macrophage polarization-associated inflammatory diseases.

### Limitations

Some limitations should be mentioned. On the one hand, this study is still based on rats and needs to be further determined in human specimens. On the other hand, the vitro experiments need to be further verified, which is also the direction of our next research.

## Conclusions

In summary, our results support the notion that lncRNA MEG8 can sponge miRNA-181a-5p to regulate SHP2 expression, thus promoting M1 macrophage polarization and suppressing JAK2/STAT3 pathway. Therefore, lncRNA MEG8 may serve as a promising therapeutic target for HSP.

## Data Availability

The data is available for reproduction of results on request from the corresponding author.

## Abbreviations

MEG8: maternally expressed gene 8
IFN-γ: Interferon γ
TGF‐β: transforming growth factor β
GAPDH: glyceraldehyde 3-phosphate dehydrogenase
JAK2: Janus kinase 2
STAT3: signal transducer and activator of transcription 3
mRNA: messenger RNA
ceRNA: competing endogenous RNAs
NC: negative control
RT-qPCR: quantitative reverse transcription polymerase chain reaction
siRNA: small interfering RNA
SHP2: the Src homology phosphotyrosylphosphatase 2
UTR: untranslated region
WT: wild type
